# Impact of Comingled Heterospecific Assemblages on Developmentally Based Estimates of the Post-Mortem Interval—A Study with *Lucilia sericata* (Meigen), *Phormia regina* (Meigen) and *Calliphora vicina* Robineau-Desvoidy (Diptera: Calliphoridae)

**DOI:** 10.3390/insects12040280

**Published:** 2021-03-25

**Authors:** Krystal R. Hans, Sherah L. Vanlaerhoven

**Affiliations:** 1 Department of Biology, University of Windsor, 401 Sunset Ave, Windsor, ON N9B 3P4, Canada; vanlaerh@uwindsor.ca; 2 Department of Entomology, Purdue University, 901 W. State St., West Lafayette, IN 47907, USA

**Keywords:** medico-legal entomology, time of colonization, accumulated degree day estimates, length-weight estimates, species interactions

## Abstract

**Simple Summary:**

In forensic entomology, blow flies are often the first insects to arrive to decomposing remains. The development rates of blow flies are used to estimate the minimum amount of time between death and discovery of the remains, or post-mortem interval (PMI). When there are multiple species of flies interacting on the same remains, there could be changes in the development of the flies. We tested the development of three different species of blow flies in different combinations at different temperatures and measured the development and the rate of growth. One species (*Lucilia sericata*) grew larger when it developed with the species *Phormia regina* at certain temperatures. The larvae of *Calliphora vicina* gained weight slower when interacting with *P. regina* and *P. regina* grew faster when interacting with these two other species. Due to these differences in the development rates of the flies, depending on the species they are interacting with, more research is needed to further examine other species combinations and temperatures.

**Abstract:**

Estimates of the minimum post-mortem interval (mPMI) using the development rate of blow flies (Diptera: Calliphoridae) are common in modern forensic entomology casework. These estimates are based on single species developing in the absence of heterospecific interactions. Yet, in real-world situations, it is not uncommon to have 2 or more blow fly species developing on a body. Species interactions have the potential to change the acceptance of resources as suitable for oviposition, the timing of oviposition, growth rate, size and development time of immature stages, as well as impacting the survival of immature stages to reach adult. This study measured larval development and growth rate of the blow flies *Lucilia sericata* (Meigen, 1826), *Phormia regina* (Meigen, 1826) and *Calliphora vicina* Robineau-Desvoidy (Diptera: Calliphoridae) over five constant temperatures (15, 20, 25, 30, 35 °C), in the presence of conspecifics or two-species heterospecific assemblages. Temperature and species treatment interacted such that *L. sericata* larvae gained mass more rapidly when in the presence of *P. regina* at 20 and 30 °C, however only developed faster at first instar. At later stages, the presence of *P. regina* slowed development of *L. sericata* immatures. Development time of *C. vicina* immatures was not affected by the presence of *P. regina*, however larvae gained mass more slowly. Development time of *P. regina* immatures was faster in the presence of either *L. sericata* or *C. vicina* until third instar, at which point, the presence of *L. sericata* was neutral whereas *C. vicina* negatively impacted development time. *Phormia regina* larvae gained mass more rapidly in the presence of *L. sericata* at 20 °C but were negatively impacted at 25 °C by the presence of either *L. sericata* or *C. vicina*. The results of this study indicate that metrics such as development time or larval mass used for estimating mPMI with blow flies are impacted by the presence of comingled heterospecific blow fly assemblages. As the effects of heterospecific assemblages are not uniformly positive or negative between stages, temperatures or species combinations, more research into these effects is vital. Until then, caution should be used when estimating mPMI in cases with multiple blow fly species interacting on a body.

## 1. Introduction

To estimate the minimum Post-mortem Interval (mPMI) using insect evidence, there are two general approaches; one method uses the predictable rates of development of blow flies (Diptera: Calliphoridae) [1,2,3] and the other uses the predictable changes in community composition in the succession of insects through decomposition [4,5]. Blow fly species have different growth and developmental rates, which have been measured for numerous species including: *Lucilia sericata* Meigen [6,7,8,9,10,11], *Calliphora vicina* Robineau-Desvoidy [6,8,10,12,13], and *Phormia regina* Meigen [6,8,14,15]. Using these species-specific development rates, combined with temperature conditions that the larvae experience during development, the mPMI estimate can be calculated. Numerous studies provide examples of variable development within species of blow flies. This may be a result of different methods such as food source, density, constant versus fluctuating temperatures, or different geographic populations [7,11,16,17,18]. Greater awareness of this issue is being addressed in the forensic entomology community through validation studies that compare estimates of mPMI using different developmental data [19].

Developmentally based estimates of mPMI must first determine how far along the insect evidence has progressed through immature larval development. This is done by examining the larval spiracular slits for instar determination [2,20], and/or by measuring larval size using either length or weight [3,21]. Any factors that impact the timing of larval development between instars or impact the rate at which larvae grow or gain mass will impact mPMI estimates based on these metrics. It is well known that growth rate, larval weight and adult size may all be influenced by temperature [22] and density of larvae on the food source [23,24,25]. Less well studied is the effect of species interactions, including competition, which can alter body size and fecundity of blow flies [25,26,27,28,29,30]. Yet, understanding the role of species interactions and temperature on the behavior and development of blow flies should allow for more accurate estimates of mPMI [31].

Although many development studies have examined the influence of temperature on blow flies, many of these studies examine only conspecifics developing, with species interactions noticeably absent [14,15,32,33,34] and it is likely that species interactions together with environmental effects result in variable blow fly development. Group oviposition sets up a potential scenario for different species interactions among larvae, such as intraspecific and interspecific competition [24,35,36]. The blow flies *L. sericata*, *C. vicina* and *P. regina* are three widely distributed species that are frequently encountered on decomposing remains [37]. These blow fly species often arrive to carrion and oviposit large aggregations of eggs. *Lucilia sericata* and *P. regina* have the same developmental range of 10–35 °C [14,38,39,40], and often colonize the same carrion resource [41,42,43,44]. *Calliphora vicina* has a colder developmental range of between 3.5–30 °C [13,45]. In southern Ontario (Canada), *C. vicina* and *P. regina* co-occur during the spring and late fall, whereas *L. sericata* and *P. regina* co-occur during the late spring through to early fall (VanLaerhoven, personal observations).

It is generally believed that *L. sericata* is a poor competitor, exhibiting negative effects such as reduced body size and reduced survival due to intraspecific competition [35,46,47]. However, when competing with *P. regina*, *L. sericata* development and survival were not reduced, but *P. regina* larvae were completely eliminated from resources when competing with *L. sericata* [48]. 

The growth rate of *C. vicina* is often greater than that of other species, due to the larger size of this species [35]. This species experiences increased mortality and reduced adult size due to intraspecific competition during development, indicating that the growth of *C. vicina* should be reduced during intraspecific interactions [24,35]. Due to their large size, *C. vicina* may not be as heavily impacted by interspecific competition when competing with smaller larvae and may have the ability to outcompete and exclude a smaller species. Conversely, the larger size of *C. vicina* may result in increased competition with other species due to their greater resource requirements.

The objective of this study was to measure the effect of conspecific or heterospecifics rearing conditions at five different constant temperatures between 15–35 °C on development time and growth rate of the blow flies *L. sericata*, *C. vicina* and *P. regina*. 

## 2. Materials and Methods

### 2.1. Colony Maintenance

All adult flies were maintained in colonies at the University of Windsor and were housed in 46 × 46 × 46 cm aluminum cages (Bioquip 1450C aluminum collapsible cage) at 25 °C, 60% RH and a 12:12 L:D diel cycle. Colonies originated from wild-caught females, collected in Windsor, Ontario, Canada with new wild type flies trapped and added to the colonies every year. Adult flies were provided with sugar and water ad libitum. Pork liver was used as an oviposition substrate for gravid females within the colony cages. Larvae were given fresh liver throughout development and were monitored until emergence of adults. After emergence, flies were transferred to clean colony cages. 

### 2.2. Experimental Design

The species treatments for this study were as follows: (1) *P. regina* only, (2) *L. sericata* only, (3) *C. vicina* only, and (4) *P. regina* and *L. sericata* mixed (5) *P. regina* and *C. vicina* mixed. In treatments 1–3, each species developed with conspecifics and therefore experienced intraspecific interactions only. Treatments 4–5 represented mixed species treatment, with the species experiencing both intra and interspecific interactions. All species and temperature treatments were replicated five times. Prior to setting up each treatment replicate, colony cages of adult flies were supplied with pork liver as an oviposition media to obtain egg masses of up to 2000 eggs. Eggs were held at 25 °C until eclosion, at which point, they were transferred to treatment conditions within an hour of eclosion. For each temperature and species treatment, 40 cups were prepared and were composed of 20 first stage larvae were transferred to 59 mL polystyrene cups using a dampened paintbrush. Density of larvae was maintained at 20 individuals regardless of treatment, thus in mixed species treatments, 10 individuals of each species were placed into the cup. Each cup contained 20 g of pork liver to ensure that excess liver would be present, as each larva requires between 0.5–1 g liver [49,50] and 1.5 cm of sawdust, to act as a pupation medium. All cups were covered with landscape tarp and secured with a plastic lid. Cups were placed into growth chambers (Conviron Adaptis A1000) that were programmed to a constant temperature, with 50% (±0.41–2.59) RH and a 12:12 L:D cycle. Temperatures within the growth chambers were programmed to be one of five temperatures (15 °C, 20 °C, 25 °C, 30 °C, 35 °C (±0.66)). Dataloggers (HOBO U-12 data loggers, Onset, Pocasset, MA, USA) were placed into the growth chambers to record hourly temperature and relative humidity.

### 2.3. Sampling

For development up to pupation, cups were checked every 8–12 h for developmental stage based on the number of spiracular slits or visual observation of pupation. Following pupation, cups were checked every 20–24 h for adult emergence. At each check, one cup from each species and temperature treatment was removed entirely from the growth chambers. All larvae present in that cup were immediately placed into boiling water for 30 s to prevent shrinkage and were then preserved in 70% ethanol. This continued until pupation was observed for all larvae in the polystyrene cups. In heterospecific treatments, larvae were identified to species using the peritreme and accessory oral sclerite [51] prior to determination of stage or larval mass. The wet mass of individual larvae was measured to the nearest 0.1 mg using an analytical scale (Denver Instruments M-120). 

### 2.4. Statistical Analyses

Analyses were conducted for each mixed species combination (*L. sericata* with *P. regina*, *C. vicina* with *P. regina*, *P. regina* with *L. sericata* and *P. regina* with *C. vicina*) to determine if species composition influenced growth rate, minimum development time and pupal mass. To satisfy the assumptions of normality and homogeneity of variance for ANOVA, variables were square-root (larval weight) transformed.

Given the strong known effect of temperature on development time, instead of a two-way ANOVA to analyze interactions between temperature and species composition treatment, Bonferonni corrected t-tests were used to compare individual pairwise comparisons within stage and temperature combinations that differed between of a species on its own compared to development time in the presence of the second species. Thus, α = 0.003 was used to distinguish significant differences.

To analyze if growth rate (mg larval mass/sampling time) differed between larvae in conspecific or heterospecific conditions, the relationship of larval mass over time was transformed into a linear relationship for each species treatment at each temperature using the equation:

HM = wH+ k
(1)

HM = product of duration of growth (in hours) and larval mass (in mg)
k = y intercept or cumulative mass acquisition during development
w = rate of change in mass or slope
H = duration of growth (in hours)

Within each temperature and pairwise species treatment of conspecific to heterospecific, an indicator variable linear regression with interaction analysis was conducted to determine if slope of the conspecific treatment regression differs from that of the heterospecific treatment regression (indicated by an interaction term with *p* ≤ 0.05). In addition, evidence of an overall mean difference between conspecific and heterospecific growth rate is indicated by a significant species treatment term (*p* ≤ 0.05).

## 3. Results

### 3.1. Development Time

As expected, temperature affected the minimum development time to each stage of all three species in this study, with faster development times as temperature increased (Table 1, Table 2 and Table 3). *Phormia regina* took less time to develop at all temperatures tested when compared to *L. sericata* and *C. vicina*, whether developing with conspecifics or heterospecifics. 

The presence of *p. regina* reduced the developmental time of *L. sericata* compared to developing with conspecifics for the first larval stages at some temperatures, but increased the developmental time of *L. sericata* for the second, third and pupal stages at some temperatures (Table 1). Development time to second instar was shorter when developing with *P. regina* than when developing with conspecifics at 20 and 30 °C with development time either not different or not significantly shorter but trending shorter at other temperatures. In contrast, development time to subsequent stages was longer when *L. sericata* developed with *P. regina*, particularly for time to third instar at 25 °C, time to pupation at 15 °C and time to adult emergence at 25 °C. Development time between *L. sericata* raised alone or with *P. regina* either did not differ or was not significantly longer but trending longer at other stages and temperatures for those reared with *P. regina*.

The presence of *L. sericata* reduced the developmental time of *P. regina* compared to developing with conspecifics for the first and second larval stages at some temperatures (Table 2). Development time to second instar was shorter when developing with *L. sericata* than when developing with conspecifics, except at 30 °C. Development time to third instar was only shorter when developing with *L. sericata* than when developing with conspecifics at 15 °C. Development time to pupal and adult emergence did not differ between those *P. regina* with conspecifics compared to those with *L. sericata*.

The presence of *C. vicina* reduced the developmental time of *P. regina* compared to developing with conspecifics for the first and second larval stages at some temperatures (Table 2). Development time to second and third instar was shorter when *P. regina* developed with *C. vicina* than when developing with conspecifics at 15 and 25 °C, and either not different or not significantly shorter but trending shorter at other temperatures. Development time to pupation was only longer when *P. regina* developed with *C. vicina* than when developing with conspecifics at 20 °C. Development time to adult emergence was longer for *P. regina* developing with *C. vicina* compared to those with conspecifics at 15–25 °C and not significantly longer but trending longer at 30 and 35 °C.

The presence of *P. regina* had no effect on the developmental time of *C. vicina* compared to developing with conspecifics at any stage or temperature (Table 3).

### 3.2. Growth Rate 

**Figure 1:**
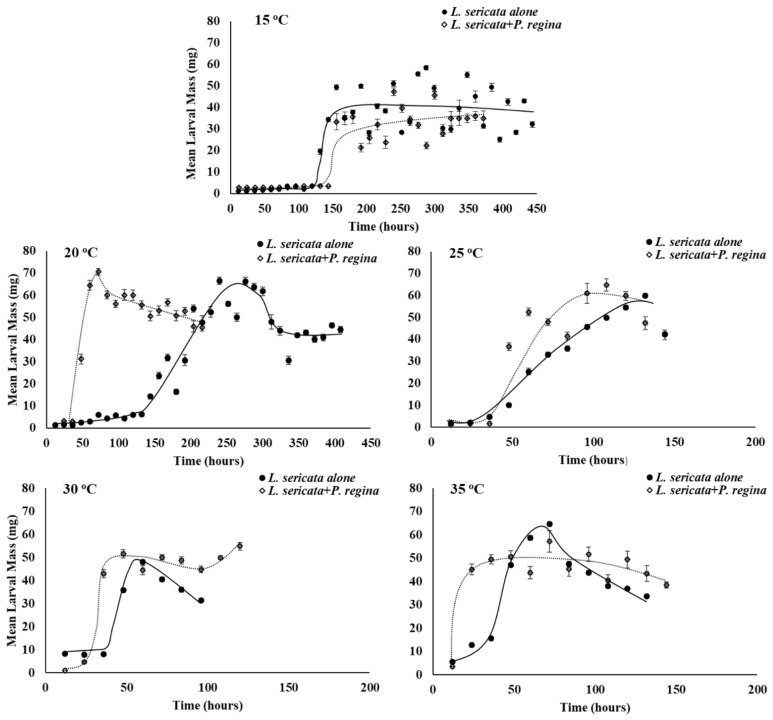
Mean (±SE) larval growth rate (mg/time) of *L. sericata* larvae reared with conspecifics, or reared with *P. regina* larvae at various temperatures. Indicator variable linear regression with interaction was conducted on the product of larval mass (mg) and time (in h) over time (not shown) for each species treatment and temperature combination. Slope (growth rate) differs at 30 °C (r^2^ = 0.96, F_1,14_ = 4.6, *p* = 0.05). Overall growth rate differs at 20 and 30 °C (r^2^ = 0.86, F_1,48_ = 6.7, *p* = 0.01; r^2^ = 0.96, F_1,14_ = 10.8, *p* = 0.005, respectively).

**Figure 2:**
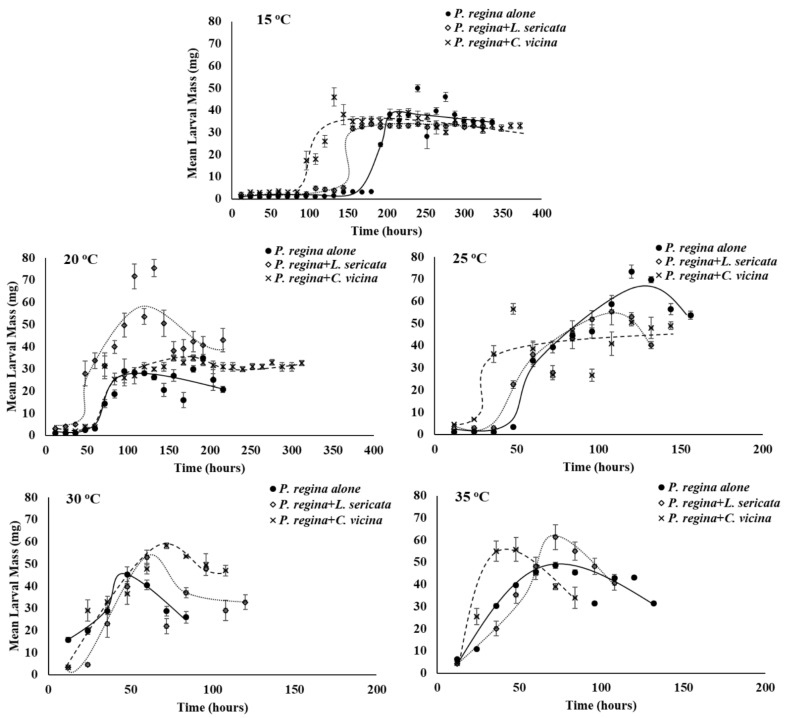
Mean (±SE) larval growth rate (mg/time) of *P. regina* larvae reared with conspecifics, or reared with *L. sericata or C. vicina* larvae at various temperatures. Indicator variable linear regression with interaction was conducted on the product of larval mass (mg) and time (in h) over time (not shown) for each species treatment and temperature combination. Slope (growth rate) differs at 25 °C for *P. regina* alone (F_2,30_ = 4.3, *p* = 0.02) and at 30 °C for *P. regina* with *C. vicina* (r^2^ = 0.89, F_2,20_ = 3.7, *p* = 0.04). Overall growth rate differs at 20 °C for *P. regina* with *L. sericata* (r^2^ = 0.89, F_2,56_ = 24.2, *p* < 0.0001) and at 30 °C for *P. regina* with *C. vicina* (r^2^ = 0.89, F_2,20_ = 5.4, *p* = 0.01).

**Figure 3:**
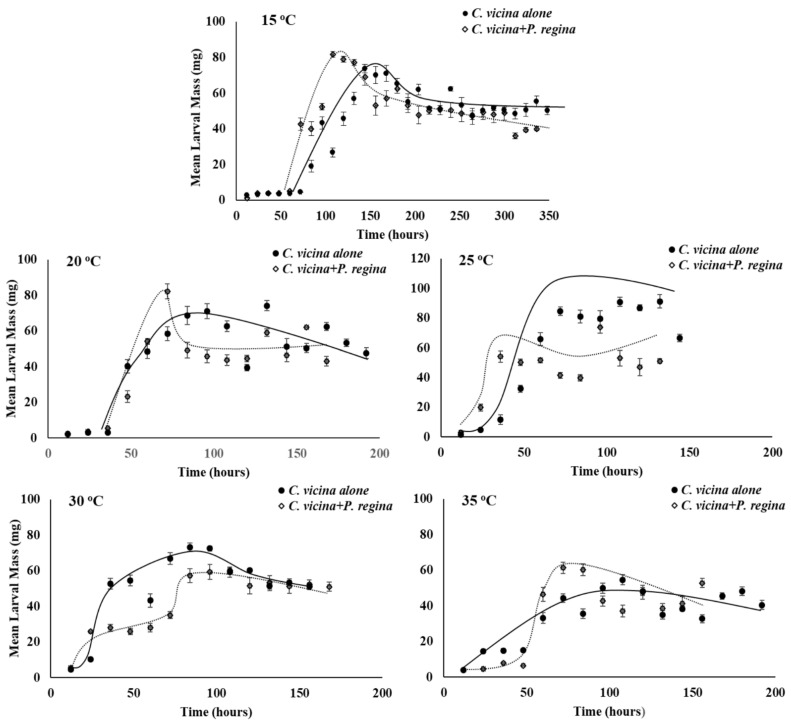
Mean (±SE) larval growth rate (mg/time) of *C. vicina* larvae reared with conspecifics, or reared with *P. regina* larvae at various temperatures. Indicator variable linear regression with interaction was conducted on the product of larval mass (mg) and time (in h) over time (not shown) for each species treatment and temperature combination. Slope (growth rate) differs at 15 and 25 °C (r^2^ = 0.89, F_1,53_ = 6.46, *p* = 0.01; r^2^ = 0.93, F_1,19_ = 13.5, *p* = 0.002, respectively). Overall growth rate differs at 25 and 30 °C (r^2^ = 0.93, F_1,19_ = 14.1, *p* = 0.001; r^2^ = 0.94, F_1,23_ = 5.44, *p* = 0.03, respectively).

As expected, temperature affected growth rate (larval mass/time) of all three species in this study, with faster mass acquisition as temperature increased through larval stages until the start of the post-feeding period prior to pupation (Figure 1, Figure 2 and Figure 3).

The slope of the regression line for larval growth rate of *L. sericata* differs between developing on its own compared to with *P. regina* at 30 °C such that *L. sericata* is slower in gaining mass over time when developing on its own (Figure 1; r^2^ = 0.96, F_1,14_ = 4.6, *p* = 0.05). In addition, *L. sericata* acquires less overall mass when on its own compared to with *P. regina* at 20 and 30 °C (r^2^ = 0.86, F_1,48_ = 6.7, *p* = 0.01; r^2^ = 0.96, F_1,14_ = 10.8, *p* = 0.005, respectively).

At 20 °C, *P. regina* acquires more overall mass when with *L. sericata* than when on its own or with *C. vicina* (r^2^ = 0.89, F_2,56_ = 24.2, *p* < 0.0001). At 25 °C, larval growth rate of *P. regina* differs between developing on its own compared to with *L. sericata* or *C. vicina* at 25 °C such that *P. regina* is initially slower in gaining mass but as development progresses, becomes faster when developing on its own (Figure 2; r^2^ = 0.92, F_2,30_ = 4.3, *p* = 0.02). In addition, at 30 °C, both the slope of the regression line and the overall growth rate differ such that *P. regina* larvae developing with *C. vicina* gain more overall mass at a faster rate than *P. regina* alone or with *L. sericata* (r^2^ = 0.89, slope: F_2,20_ = 3.7, *p* = 0.04, overall: F_2,20_ = 5.4, *p* = 0.01).

The slope of the regression line for larval growth rate of *C. vicina* differs between developing on its own compared to with *P. regina* at 15 and 25 °C such that *C. vicina* is initially slower at gaining mass when developing on its own but becomes faster when developing on its own over time (Figure 3; r^2^ = 0.89, F_1,53_ = 6.46, *p* = 0.01; r^2^ = 0.93, F_1,19_ = 13.5, *p* = 0.002, respectively). In addition, *C. vicina* acquires more overall mass when on its own compared to with *P. regina* at 25 and 30 °C (r^2^ = 0.93, F_1,19_ = 14.1, *p* = 0.001; r^2^ = 0.94, F_1,23_ = 5.44, *p* = 0.03, respectively).

## 4. Discussion

This study has demonstrated that metrics such as development time or larval mass used for estimating mPMI with blow flies are impacted by the presence of comingled heterospecific blow fly assemblages and the effects are not uniformly positive or negative between stages, temperatures or species combinations (Table 4). Indeed, this complexity in response to heterospecifics is expected as co-existence of these three blow fly species within overlapping temporal and spatial scales requires evolution of fluctuation dependent and independent stabilizing mechanisms such as facilitation, environmental fluctuation and resource partitioning [52,53]. Similarly, it has been argued that larval aggregation may be costly or beneficial [54] but it is expected that the outcome depends on the specific species, stages, densities, amount of resources and temperatures [52].

Previous studies have found that *L. sericata* is a poor competitor, by displaying negative effects on survival and adult size due to interspecific competition [35,46,47]. When reared together with *C. vicina*, *L. sericata* developed slower than when developing with conspecifics at 25 °C [55]. It has been suggested that costs of interspecific aggregation may be from generating temperatures closer to thermal maximum of a species, decreased quantity of food resource and availability of nutrients, and increased risk of pathogens and disease [54]. 

Other studies have indicated that *L. sericata* may be facilitated by the presence of other species, such as *P. regina* [56]. In our study, temperature and species treatment interacted such that *L. sericata* larvae gained mass more rapidly when in the presence of *P. regina* at 20 and 30 °C, providing support for the idea that *L. sericata* may benefit by the presence of *P. regina.* Indeed, *L. sericata* also developed faster with *P. regina*, but only during the first instar. However, at later stages the presence of *P. regina* slowed development of *L. sericata* immatures. In studies with *C. vicina*, *L. sericata* pupae were larger when reared with heterospecifics at 15 °C [57] and at 25 °C [55]. In a study by Fouche et al. [58], *L. sericata* adult size was larger when larvae were reared with *C. vicina*, than when reared separately at 25 °C on 7 day rotted liver but not on fresh liver. Similarly, *L. sericata* had reduced mortality when developing with *C. vicina*, than when developing with conspecifics at 25 °C [55] but not at 15 or 28 °C [57]. 

*Lucilia sericata* demonstrates considerable plasticity in growth and development [17,55,59,60] and adaptations to combat adverse conditions, either environmental or due to species interactions, consist of rapid growth during larval stages to reach their critical weight for pupation in order to promote greater survival [17,59]. Developmental plasticity is also observed in *C. vicina*, when developing in heterospecific combinations with *L. sericata*, developed faster and migrated earlier at higher temperatures [57,60]. 

At temperatures above 30 °C, Reiter [12] reported that *C. vicina* larvae exhibit inhibited growth, with high mortality and few surviving to pupation. This is not surprising given that *C. vicina* is considered a cold weather species [2,13]. At 35 °C, *C. vicina* did not successfully emerge from pupation regardless of species treatment. However, when developing with *P. regina*, *C. vicina* larvae failed to successfully emerge from pupation at 30 °C, an indication that *P. regina* negatively impacted *C. vicina* at this temperature. Given that development time of *C. vicina* immatures was not affected by the presence of *P. regina*, it is possible that temperature and the presence of *P. regina* interacted to reduce the ability of *C. vicina* to achieve its critical weight for successful pupation and emergence to adult. 

Critical weights required for pupation have been studied in many insect systems [61,62,63]. Saunders and Bee [24] found that the minimum pupal weight for *C. vicina* was 30 mg when developing in low densities of less than 50 larvae, but this critical weight was reduced to 15–20 mg when *C. vicina* was developing at higher densities of 150 conspecific larvae or more. These results indicate that critical weight may fluctuate due to the influence of species interactions and competition. In this study, *C. vicina* larvae gained mass more slowly in the presence of *P. regina*, which may explain the lack of survival of *C. vicina* to adult emergence at 30 and 35 °C. 

The presence of proteolytic enzymes released by *L. sericata* may facilitate more efficient feeding by other blow fly species [25,30,47,64]. Scanvion et al. [65] found that exodigestion with enzymes aided in the consumption of the carrion resource and enhanced development of *L. sericata*. When developing in higher densities, *L. sericata* developed faster and had decreased mortality, due to enzymatic activity [65]. Development time of *P. regina* immatures was faster in the presence of either *L. sericata* or *C. vicina* until third instar, at which point, the presence of *L. sericata* was neutral whereas *C. vicina* negatively impacted development time. The release of these enzymes may be a mechanism that facilitates feeding by *P. regina*, resulting in larger larvae at some temperatures and faster initial development. 

Temperature can also have an effect, as there is a direct relationship between temperature and feeding rates; as temperatures increase, feeding rates increase and larvae may not be able to metabolize as quickly, leading to smaller individuals [17,66]. This may explain why *P. regina* larval growth rate was negatively impacted at 25 °C by the presence of either *L. sericata* or *C. vicina* but *P. regina* larvae gained mass more rapidly in the presence of *L. sericata* at 20 °C.

## 5. Conclusions

As presented in this study, there is complexity to understanding the impact of species interaction on the metrics used to estimate immature development, whether it be growth rate measured by larval mass or length over time, or time to each developmental stage. Clearly it depends on the particular species involved and the temperatures.

Fluctuating temperatures exemplify temperatures blow flies experience in natural conditions, but the results of fluctuating temperatures on larval development are mixed. Some species develop faster under fluctuating temperatures, such as *Calliphora vomitoria* L. [67], whereas development of *L. sericata*, *P. regina* and *C. vicina* are delayed under periods of fluctuating temperatures [8,68]. In addition, larvae may balance thermoregulation and social behavior, trading off aggregation in suboptimal temperatures with thermal optimization [69]. Since temperature interacts with development to mediate species interactions, fluctuating temperatures have the potential to change outcomes of species interactions.

It is likely that density of individuals will impact these outcomes. The density of 20 larvae utilized in this study limits the amount of competition/facilitation that the larvae can experience while developing. On carrion, much higher densities of larvae occur which is influenced by the recruitment of gravid females and larval aggregation behavior. Benefits of aggregation include increased heat production, enzyme activity and cooperative feeding, reduced risk of predation and parasitism and protection from fluctuations in environmental factors which overall may result in faster development and increased survival [3,54,70]. However, as density of individuals increases, the cost associated with aggregation are generating temperatures closer to thermal maximum of a species, decreased quantity of food resource and availability of nutrients, and increased risk of pathogens and disease which may result in smaller sizes, decreased weight and decreased survival of the species involved [54]. Presumably, this would also exacerbate species interactions. 

However, it is not only the overall density that is important. It is likely that initial densities of each species’ population affect the outcome of the interaction, where the more abundant species has the greatest probability of dominating a resource [52,71]. Known as founder control, at each carrion resource, a different species could arrive to and colonize the resource at a greater abundance ultimately resulting in different potential outcomes of species interactions on each of these carrion patches [52]. 

Ultimately, research that incorporates species interactions and ecological theory should allow forensic entomologists to better model insect development for use in estimating mPMI. As we account for more of the factors affecting insect development of each forensically relevant species we will improve our estimates, thereby increasing confidence in the interpretation of insect evidence in the judicial system [31].

## Figures and Tables

**Table 1:** Mean minimum development times (h) to reach each life stage starting from 1 h old first instar larvae for *L. sericata* when developing with conspecifics (*L. sericata* alone) and heterospecifics (*L. sericata* with *P. regina*). Variation around the mean is presented as the greater of either half the sampling interval (6 h for second instar to pupal stages, 12 h for adult stage) or the calculated standard error. Asterisk * indicate differences (*p* < 0.003) between *L. sericata* alone or with *P. regina* combinations within a single stage and temperature pairwise comparison.

Species	*L. sericata* Alone				*L. sericata* with *P. regina*			
Temp (°C)	L2	L3	Pupae	Adult	L2	L3	Pupae	Adult
15	92 ± 6	125 ± 6	437 ± 6	1119 ± 12	87 ± 6	147 ± 16	498 ± 30 *	1122 ± 55
20	49 ± 6	65 ± 6	221 ± 6	533 ± 12	24 ± 6 *	72 ± 6	228 ± 17	524 ± 30
25	16 ± 6	30 ± 6	158 ± 9	325 ±12	16 ± 6	61 ± 6 *	139 ± 6	385 ± 12 *
30	25 ± 6	49 ± 6	111 ± 6	236 ± 12	16 ± 6 *	35 ± 6	128 ±8	282 ± 17
35	17 ± 6	32 ± 6	145 ± 10	248 ±18	12 ± 6	30 ± 6	138 ± 12	244 ± 12

**Table 2:** Mean minimum development times (h) to reach each life stage starting from 1 h old first instar larvae for *P. regina* when developing with conspecifics (*P. regina* alone) and heterospecifics (*P. regina* with *L. sericata* or with *C. vicina*). Variation around the mean is presented as the greater of either half the sampling interval (6 h for second instar to pupal stages, 12 h for adult stage) or the calculated standard error. Asterisk * indicate differences (*p* ≤ 0.003) between *P. regina* alone and with *L. sericata* combinations, or with *C. vicina* combinations within a single stage and temperature pairwise comparison.

Species	*P. regina* Alone				*P. regina* with *L. sericata*				*P. regina* with *C. vicina*			
Temp (°C)	L2	L3	Pupae	Adult	L2	L3	Pupae	Adult	L2	L3	Pupae	Adult
15	156 ± 7	209 ± 6	439 ± 42	869 ± 51	87 ± 6 *	147 ± 16 *	488 ± 40	972 ± 47	26 ± 6 *	101 ± 6 *	370 ± 6	1202 ± 12 *
20	51 ± 6	73 ± 6	217 ± 6	409 ± 12	24 ± 6 *	65 ± 6	212 ± 10	420 ± 20	43 ± 6	72 ± 6	298 ± 10 *	537 ± 27 *
25	50 ± 6	65 ± 6	139 ± 6	253 ± 12	16 ± 6 *	61 ± 6	133 ± 6	259 ± 12	24 ± 6 *	48 ± 6*	158 ± 6	277 ± 12 *
30	19 ± 6	35 ± 6	95 ± 6	201 ± 12	16 ± 6	35 ± 6	121 ± 10	227 ± 12	12 ± 6	24 ± 6	96 ± 6	216 ± 12
35	16 ± 6	31 ± 6	93 ± 6	174 ± 12	10 ± 6 *	27 ± 6	99 ± 6	195 ± 12	12 ± 6	24 ± 6	86 ± 10	182 ± 12

**Table 3:** Mean minimum development times (h) to reach each life stage starting from 1 h old first instar larvae for *C. vicina* when developing with conspecifics (*C. vicina* alone) and heterospecifics (*C. vicina* with *P. regina*). Variation around the mean is presented as the greater of either half the sampling interval (6 h for second instar to pupal stages, 12 h for adult stage) or the calculated standard error. N/A indicates all individuals died prior to reaching this stage.

Species	*C. vicina* alone				*C. vicina* with *P. regina*			
Temp (°C)	L2	L3	Pupae	Adult	L2	L3	Pupae	Adult
15	33 ± 6	78 ± 6	342 ± 6	798 ± 12	26 ± 6	72 ± 6	322 ± 6	787 ± 12
20	15 ± 6	53 ± 6	197 ± 6	505 ± 12	23 ± 6	53 ± 6	178 ± 6	432 ± 25
25	15 ± 6	27 ± 6	140 ± 8	413 ± 13	12 ± 6	24 ± 6	130 ± 10	451 ± 12
30	12 ± 6	24 ± 6	154 ± 6	366 ± 12	12 ± 6	24 ± 6	146 ± 6	N/A
35	29 ± 6	50 ± 6	216 ± 6	N/A	26 ± 6	55 ± 6	124 ± 33	N/A

**Table 4:** Summary of heterospecific species treatment effects on development time and growth rate (larval mass/time) when compared to species reared alone. + indicates a positive effect; − indicates a negative effect; = indicates a neutral effect.

Species Treatment	Development Time	Growth Rate
*L. sericata* with *P. regina*	+ until 2nd instar, particularly at 20 & 30 °C − 2nd instar onward	+ at 20 & 30 °C
*C. vicina* with *P. regina*	=	−
*P. regina* with *L. sericata*	+ until 3rd instar = 3rd instar onward	+ at 20 °C − at 25 °C
*P. regina* with *C. vicina*	+ until 3rd instar − 3rd instar onward	− at 25 °C

## Data Availability

The data presented in this study are openly available in FigShare at 10.6084/m9.figshare.14294699.
